# Atomic scale analyses of 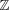-module defects in an NiZr alloy

**DOI:** 10.1107/S2053273318011439

**Published:** 2018-10-04

**Authors:** Abdullah Sirindil, Raphael Kobold, Frédéric Mompiou, Sylvie Lartigue-Korinek, Loic Perriere, Gilles Patriarche, Marianne Quiquandon, Denis Gratias

**Affiliations:** a CNRS-Laboratoire de Métallurgie de l’UMR 8247, IRCP Chimie-ParisTech, 11 rue Pierre et Marie Curie, 75005 Paris, France; b Institute of Material Physics in Space, German Aerospace Center (DLR) Linder Höhe, 51170 Cologne, Germany; c CNRS-CEMES and Université de Toulouse, 29 rue Jeanne Marvig, 31055 Toulouse, France; d CNRS-ICMPE UMR 7182 CNRS-UPEC, 2-8 rue Henri Dunant, 94320 Thiais, France; e CNRS-C2N – Marcoussis UMR 9001, Route de Nozay, 91460 Marcoussis, France

**Keywords:** {\bb Z}-module, defects, twins, dislocations, HREM-HAADF

## Abstract

Some specific structures of intermetallic alloys, like approximants of quasicrystals, have their unit cells and most of their atoms located on a periodic fraction of the nodes of a unique -module [a set of the irrational projections of the nodes of a (*N* > 3-dimensional) lattice]. Those hidden internal symmetries generate possible new kinds of defects like coherent twins, translation defects and so-called module dislocations that have already been discussed elsewhere [Quiquandon *et al.* (2016 ▸). *Acta Cryst.* A**72**, 55–61; Sirindil *et al.* (2017 ▸). *Acta Cryst.* A**73**, 427–437]. Presented here are electron microscopy observations of the orthorhombic phase NiZr – and its low-temperature monoclinic variant – which reveal the existence of such defects based on the underlying -module generated by the five vertices of the regular pentagon. New high-resolution electron microscopy (HREM) and scanning transmission electron microscopy high-angle annular dark-field (STEM-HAADF) observations demonstrate the agreement between the geometrical description of the structure in five dimensions and the experimental observations of fivefold twins and translation defects.

## Abstract 2

This article describes the observation and determination of 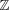-module defects (twins, translation faults and module dislocations) in NiZr by high-resolution electron microscopy (HREM), and scanning transmission electron microscopy bright-field (STEM-BF) and high-angle annular dark-field (STEM-HAADF).

## 1. Introduction   

The present article is the experimental continuation of a search to identify possible new defects in structures where the atoms, in addition to being periodically distributed, are located on a long-range-ordered subset of the nodes of a 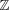-module.

The phase diagram of the binary system (Ni, Zr) presents a congruent solidification point at 1533 K for the equiatomic composition Ni_50_Zr_50_ close to a eutectic transformation, on the Zr-rich side, with a precipitation of Zr_67_Ni_33_ at 1295 K. The equiatomic Ni_50_Zr_50_ phase has an orthorhombic structure which, as will be demonstrated later, has the very remarkable property of being fully described using one unique pentagonal 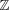-module including both atomic species. This orthorhombic stoichiometric NiZr structure was first reported by Kirk­patrick *et al.* (1962 ▸) as a CrB-type structure and has a remarkable tendency to generate quinary twins. This feature has been discussed in depth in a general framework by Parthé (1976 ▸). The first direct observations of those twins by transmission electron microscopy (TEM) were performed by Jiang *et al.* (1985 ▸) and a few years later, in much more detail, by Bouzy *et al.* (1991 ▸). Very recently, an impressive experimental study of the morphology of slowly solidified samples cooled in a containerless electrostatic levitation furnace has revealed beautiful central twins forming an almost perfect decagon in solidified spherical samples, as observed by scanning electron microscopy (SEM), energy-dispersive X-ray spectroscopy (EDS) and electron backscatter diffraction (EBSD) (Hornfeck *et al.*, 2014 ▸).

## 2. Embedding the NiZr orthorhombic (monoclinic) phase in five dimensions   

**Figure 1:**
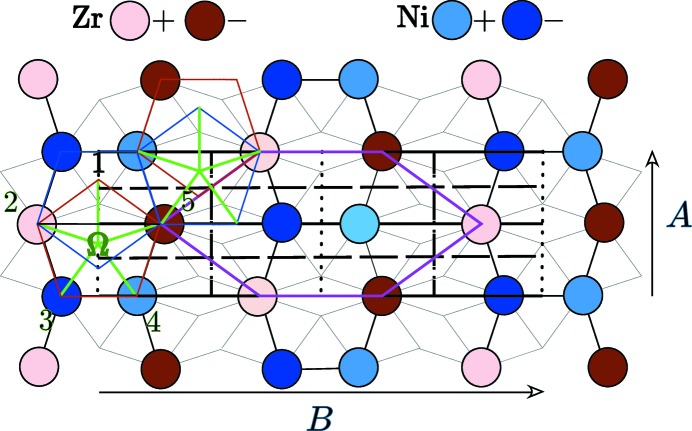
The NiZr structure (Kirkpatrick *et al.*, 1962 ▸) is a CrB-type structure *Cmcm* with lattice parameters |*A*| = 0.3268, |*B*| = 0.9973 and |*C*| = 0.4101 nm, defined by two Wyckoff positions: Ni at (0, 0.0817, 1/4) and Zr at (0, 0.3609, 1/4). Ni and Zr atoms projected in the [001] plane form hexagons that are the superimposition of two opposite regular pentagons (in red and blue in the figure) sharing the same diagonal with an accuracy better than 1%. The symbols + and − correspond to the *z* coordinates being equal, respectively, to 1/4 and −1/4. The two usual tiles of the Penrose tiling (rhombi of acute angles 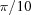 and 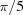) are outlined in light grey. All atomic positions belong to the 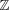-module generated by the five vectors in green noted from 1 to 5. The structure can advantageously be described as a tiling of a unique equilateral hexagonal prototile drawn in purple (see Fig. 3 ▸).

After Kirkpatrick *et al.* (1962 ▸), the orthorhombic phase NiZr has the space group *Cmcm* with lattice parameters |*A*| = 0.3268, |*B*| = 0.9973 and |*C*| = 0.4101 nm. It is defined by two Wyckoff positions 4*c* *m*2*m* (0, *y*, 1/4) with *y*_Ni_ = 0.0817 and *y*_Zr_ = 0.3609 as shown in Fig. 1 ▸.

All Ni and Zr atoms are distributed along the *z* direction at locations 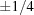. It is thus possible, with no loss of information, to characterize this *z* coordinate by a simple two-valued symbol 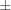 analogous to an internal scalar spin parameter.

Considering the two remaining coordinates 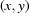, the fundamental point to emphasize is that the hexagons observed on the projection of the structure along the direction [0, 0, 1] are very close^1^ to the hexagons that are obtained by superimposing two opposite regular pentagons sharing the same diagonal^2^ as shown in Fig. 1 ▸. Because of this very specific internal geometry of the hexagons and the way they are connected in rows, the resulting projected two-dimensional structure can be entirely described using the node positions of the five-dimensional regular primitive hypercubic lattice where the five basic vectors project along the vertices of a regular pentagon. This is made clear in Fig. 1 ▸ where a tiling is drawn in the background in light grey based on the two basic prototiles (rhombi of acute angles 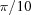 and 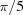) of the famous Penrose tiling (Penrose, 1979 ▸): all atoms are located on certain nodes of this tiling. Therefore, and very similarly to the case of quasicrystals (see Shechtman *et al.*, 1984 ▸), this allows us to reformulate the ideal structure of NiZr by embedding it in a five-dimensional space using atomic positions with five indices for the 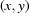 description, in addition to the scalar two-valued spin-like index 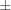 representing the *z* coordinate 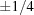. This new configurational five-dimensional Euclidian space corresponding to the 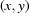 plane decomposes as
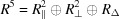
where 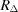 is the one-dimensional line along the main diagonal 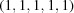 in five-dimensional space. This is the standard way of generating the Penrose tiling using the cut-and-project method [see for instance Duneau & Katz (1985 ▸), Kalugin *et al.* (1985 ▸), Elser (1986 ▸)].

The five indices are unambiguously determined up to any five-dimensional translation along the main diagonal 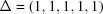. For simplicity and with no loss of generality, we choose to gather all atomic positions in a unique and the same four-dimensional plane perpendicular to Δ: the actual atomic positions *V* of NiZr can thus be expressed as *V* = 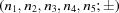, with 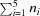 = Const.

As easily seen in Fig. 1 ▸, the two-dimensional (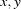) unit cell of NiZr is defined by the five-dimensional vectors *A* = 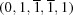 and *B* = 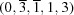, both perpendicular to Δ. Because of its *C* character, the two-dimensional lattice generated by *A* and *B* in five dimensions, say 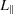, is defined by 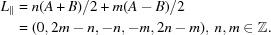
The structure itself is defined by four translation orbits^3^ satisfying the (arbitrary) condition 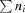 = Const. = 1, irrespective of the point symmetry elements:
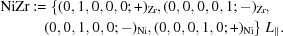
The five-dimensional symmetry elements are written as usual as 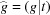 where *t* is the associated five-dimensional translation and *g* is the point symmetry operation economically written as signed permutations 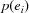 of the five unit vectors in five dimensions and a simple multiplication 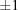 for the standard *z* coordinate: 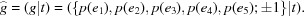



For example the *c* mirror perpendicular to *B* in *Cmcm* transforms 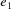 into itself, 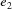 into 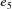 and *vice versa*, 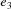 into 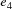 and *vice versa*, and adds 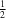 to the *z* coordinate thus transforming 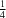 (symbol +) into 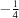 (symbol −) and *vice versa*, corresponding for the scalar component to a multiplication by −1. It can therefore be written as 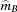 = 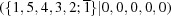 after choosing the point Ω in Fig. 1 ▸ as origin. Similarly, the mirror perpendicular to *A* can be written 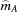 = 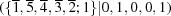. Finally, the mirror perpendicular to *C* and passing through *z* = 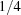 reduces to the identity in the present five-dimensional representation: 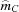 = Id = 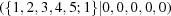.

Thus, the orthorhombic NiZr structure can be described using a subset of a Penrose tiling using the two rhombi of acute angles 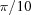 and 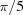. As defined in a previous article (Sirindil *et al.*, 2017 ▸) we call this kind of structure a 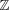-module-based alloy.

### 2.1. Elementary five-dimensional geometry   

Starting from a five-dimensional node (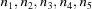), we obtain its components (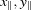) in the physical space 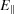 = 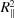 and its three components (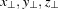) in the complementary space 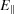 = 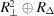 according to the following usual formulas (see, for instance, Duneau & Katz, 1985 ▸) using φ = 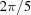: 
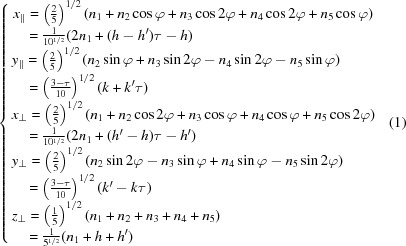
with τ = 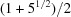, *h* = 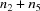, 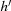 = 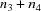, *k* = 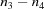, 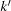 = 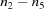 and using 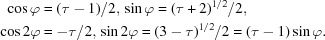
To generate the orthorhombic NiZr structure, we apply a shear of the five-dimensional lattice Λ along 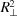 – keeping 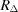 invariant – in such a way as to align two independent nodes of Λ along 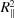 by the transformation (see Jarić & Mohanty, 1987 ▸; Gratias *et al.*, 1995 ▸): 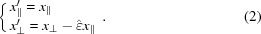
This will generate a two-dimensional lattice in 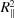. Taking *A* and *B*, the two five-dimensional vectors, the projections of which in 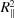 define the unit cell of the structure, we ensure the generated structure is periodic of periods 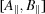 by applying the shear matrix 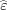 such that 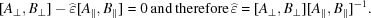
Using *A* = 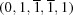 and *B* = 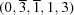, we obtain 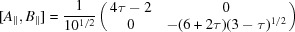
and 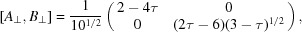
leading to 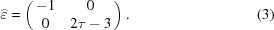
Equations (1) and (2) together with the explicit expression (3) of 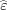 are all we need to fully handle the embedding of the NiZr three-dimensional structure into the five-dimensional superspace back and forth and generate all possible defects that keep the underlying 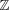-module invariant in 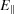.

### 2.2. Construction of the atomic surfaces generating the orthorhombic NiZr structure   

The definition of atomic surfaces for periodic structures has been discussed in a previous article (Sirindil *et al.*, 2017 ▸) where it has been shown that the simplest choice of atomic surfaces is to collect the Voronoi cells in 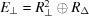 centred on the projections in 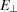 of the translation orbits defining the structure.

**Figure 2:**
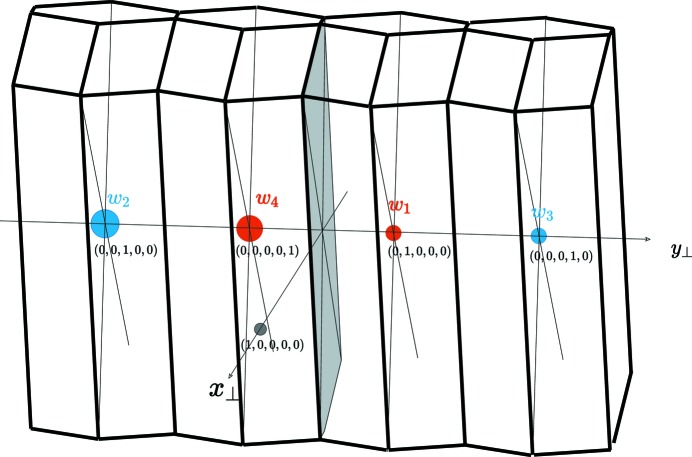
The four Voronoi cells in 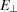 defining the atomic surfaces for the orthorhombic phase of NiZr are aligned along the 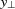 direction. This very unusual situation suggests generating the structure using the hexagonal tile based on the three translations 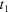 = 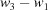 = 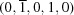, 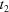 = 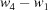 = 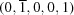 and 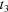 = 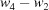 = 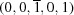, all three relating a Zr atom to another Zr atom.

Here, the orthorhombic structure is generated by four translation orbits: *w_1* = 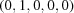, 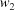 = 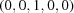, 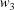 = 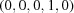 and 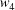 = 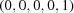 as shown in Fig. 2 ▸. Using relations (2) and (3), we note that the nodes *V* = 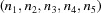 of Λ project after shear in 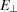 = 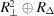 as 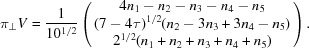
This makes the four translation orbits of the structure projecting in 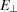 *four aligned points* along 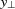 as shown in Fig. 2 ▸: 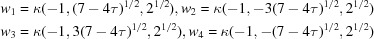
with 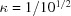.

**Figure 3:**
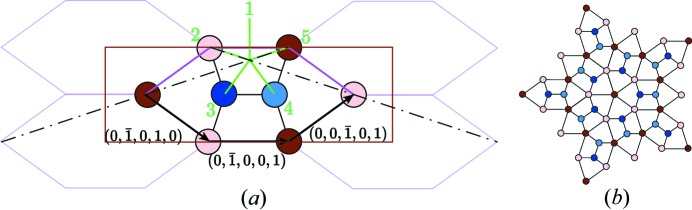
(*a*) The equilateral hexagonal prototile that generates the orthorhombic NiZr structure opens up a wide variety of possible tilings including the high-symmetry pentagonal snowflake (*b*). Whatever the tiling, all atoms apart from the unique central one in (*b*) share the same kind of environment to first neighbours as in the CrB reference structure. The set (*b*) describes the whole possible twins in the decomposition from 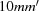 to 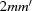: each variant can have two adjacent variants, obtained by a rotation 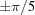 [or equivalently by a *c* mirror in the planes 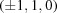].

This analysis suggests that the structure can also economically be viewed as a perfect tiling of a hexagonal prototile, as shown in Fig. 3 ▸(*a*), defined by the three vectors 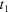 = 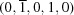, 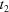 = 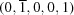 and 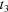 = 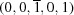 which generate the network of the Zr positions. Each of these hexagonal tiles is the very equivalent of a primitive unit cell. As will be shown later, the fact that the lengths of the three vectors 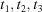 are equal means that several kinds of tiling are equally possible, as exemplified in Fig. 3 ▸(*b*), with no change in the chemical species and atomic bonds of first neighbours. *This makes twinning the easiest defect process in this alloy*.

### 2.3. Symmetry breaking: the basic defect   

**Figure 4:**
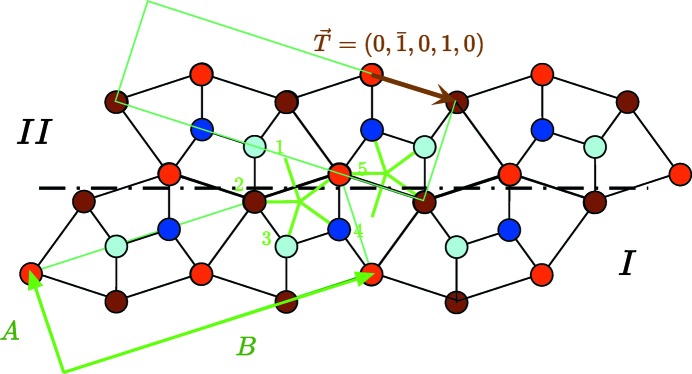
The simplest model of the structure of the quinary twin from the analysis in terms of 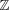-module invariance as illustrated by the two pentagons in green. The twin operation 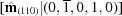 is expressed in the *Cmcm* unit-cell coordinates.

The point symmetry of the four-dimensional lattice perpendicular to Δ which generates the 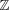-module is 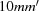 irrespective of the nature of the chemical species. Thus, the symmetry breaking induced by the perpendicular shear from 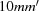 to 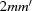 generates five twin variants issued from the coset decomposition: 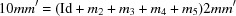
where 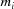 designates the mirrors containing the *i*th vector defining the pentagon (in green in Fig. 3 ▸). Because of the *c* mirror of the structure, the variants are two-by-two equivalents: the mirrors 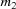 and 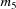 are in the 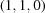 and 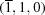 planes whereas the mirrors 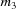 and 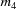 are in irrational planes with respect to the structure. Thus, a given variant can have only two different adjacent twins symmetric with respect to its *c* mirror. The atomic model of the twin operation is shown in Fig. 4 ▸. We choose the origin on the atomic Zr site number 5 in the figure. The twin point operation is the mirror defined by
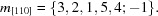
The multiplication by −1 on the spin variable corresponds to the fact that to be coherent with respect to the tiling the mirror twin must transform 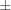 symbols into 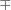 implying thus an irreducible translation along *z* by 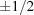.

The translation 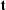 associated with the twin in the 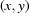 plane is shown in brown in Fig. 4 ▸ and is written 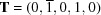. It decomposes into two parts: 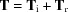 where 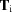 is the irreducible component independent of the choice of the origin and 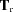 is the reducible part that vanishes by choosing the origin on the mirror:^4^

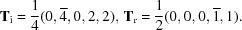
In the standard *Cmcm* unit-cell coordinates, the irreducible translation, including the component along the *z* direction, is therefore 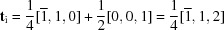
which is identical to the irreducible translation proposed several years ago by Bouzy *et al.* (1991 ▸) based on a quite different approach. Because of the *C* lattice, translation 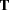 in Fig. 4 ▸ can equivalently be written as 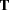 = 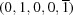 which translates into 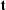 = 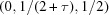 expressed on the unit cell of crystal I. As expected from the expression of the pentagonal projections (1), the *y* component of this translation is an irrational fraction of the lattice parameter *B*.

This basic mirror twin defect is remarkably coherent with respect to the atomic structure. Because the tiling of the elementary hexagons remains continuous at the level of the interface, the chemical bonds between first neighbours are respected in all aspects, chemistry, lengths and angles between bonds. This makes this defect an excellent candidate to be actually observed in real crystals as will be shown next.

**Figure 5:**
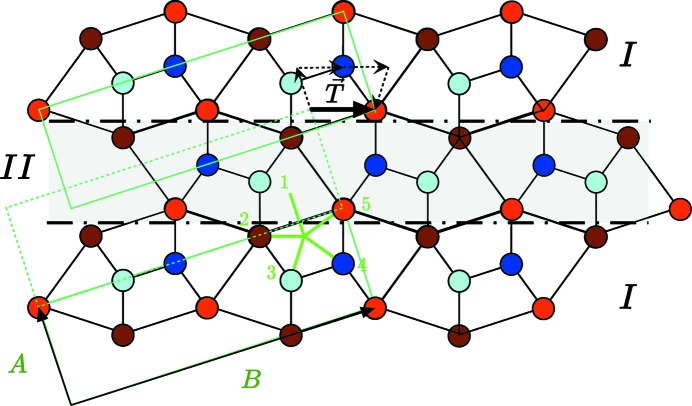
The simplest 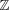-module translation defect model consists of introducing a thin slab of twinned hexagons (noted II) along a 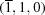 plane. This generates an elementary translation between the upper and lower parts of crystal I of **T** = 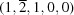 (see the fine dotted arrows) corresponding to **t** = 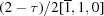 in the *Cmcm* unit cell.

Translation boundaries are easily obtained by using two consecutive mirror twin defects. The thinnest translation defect is generated by inserting one single slab of twinned equilateral hexagons as shown in Fig. 5 ▸. The associated translation is 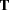 = 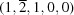 that is 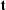 = 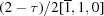 expressed in the *Cmcm* unit-cell coordinates. Of course, other global translation defects can be constructed using *N* slabs of twinned hexagons instead of one, leading thus to 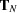 = 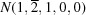 or 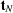 = 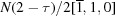.

**Figure 6:**
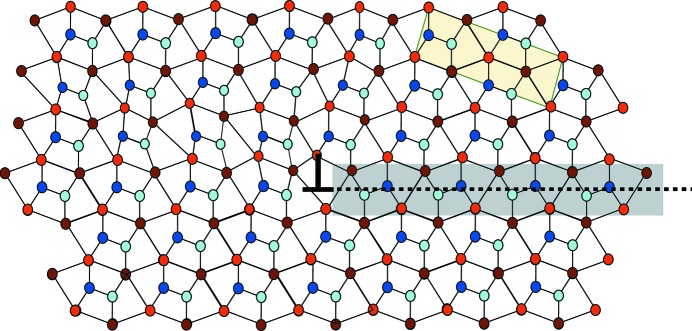
Simple sketch of the basic module dislocation with Burgers vector 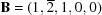.

Finally, we expect to observe the module dislocations that border the previous translation defect, *i.e.* module dislocations with a Burgers vector 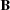 = 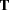 = 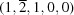 as shown in Fig. 6 ▸. This simple Burgers vector of the five-dimensional lattice takes an irrational value 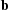 = 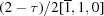 once expressed in the orthorhombic unit-cell coordinates. Observe that this dislocation is very special: as the translation defect is in fact a thin slab of a twinned crystal, the dislocation is the interface dislocation necessary to grow one step of a slab of a twinned individual in the other making it a so-called disconnection (see, for example, Hirth & Pond, 1996 ▸).

## 3. Sample preparation   

In order to check the validity of our previous predictions from the 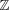-module description, we prepared samples of NiZr for high-resolution TEM investigations in high-resolution electron microscopy (HREM) and scanning transmission electron microscopy high-angle annular dark-field (STEM-HAADF) modes. Samples used in the present study were obtained by two different methods:

(*a*) A first fusion under magnetic levitation of a mother alloy of nominal atomic composition Ni_44_Zr_56_, remelted and rapidly quenched by planar flow casting and annealed for one week at 973 K in sealed ampoules under vacuum.

(*b*) High-purity elements [purity of Zr 99.97% (Smart Elements), Ni 99.995% (Alfa Aesar)] were prepared and alloyed by arc-melting at the DLR in Cologne (Germany) yielding a spherical sample of intermetallic NiZr with a diameter of 3 mm. Subsequently the sample was processed in an electrostatic levitation furnace (ESL)^5^ under high vacuum conditions (10^−5^ Pa) in order to obtain a single homogeneous crystallization event at high undercoolings (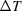 = 300 K) and specific microstructural features as described by Hornfeck *et al.* (2014 ▸).

Samples (*a*) are roughly 300 µm-thick ribbons in which small discs of 3 mm in diameter are cut and polished. The characterization by X-ray powder diffraction has been performed on a Panalytical X’PERT Pro diffractometer using Co *K*α radiation with wavelength 0.17889 nm. The powder spectra reveal that the samples contain essentially the orthorhombic phase and a few per cent in volume of NiZr_2_.

Concerning samples (*b*), since the orthorhombic phase grows in large multi-twinned grains with the [0,0,1] direction being clearly identifiable by sample surface features, these samples have been cut perpendicularly to the [0,0,1] direction and subsequently ground and polished until discs of about 150 µm remained. The highly accurate orientation of the samples makes the observations by HREM and STEM-HAADF particularly efficient: because of the strong induced texture, the orthorhombic phase grows in large multi-twinned grains with the direction [0,0,1] being the normal to the disc plane.

## 4. Electron microscopy analyses at atomic resolution   

Samples (*a*) were thinned by mechanical grinding down to 100 µm and eventually thinned to electron transparency using ion milling (Gatan PIPS) until the formation of a hole. Samples (*b*) were electropolished using a Tenupol with a solution of 10% perchloric acid and 90% methanol at 243 K under 35 V.

Both samples (*a*) and (*b*) have been examined in HREM mode on two different machines: a Topcon 002B equipped with an LaB_6_ cathode operating at 200 keV with a point-to-point resolution of 0.18 nm (ICMPE, Thiais, France) and a Hitachi 3300 electron microscope operating at 300 keV with 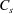 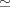 0, 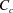 = 3.7 mm (CEMES, Toulouse, France). The STEM-HAADF and STEM bright-field (STEM-BF) observations have been performed on an FEI Titan Themis 200 [Center for Nanosciences and Nanostructures (C2N), Marcoussis, France]. This microscope uses an XFEG gun under 200 keV and is equipped with a 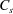 corrector (point-to-point resolution around 80 pm) and a CMOS CETA 4 k × 4 k camera. It can operate under various imaging STEM modes: BF and HAADF.

In both (*a*) and (*b*) samples, conventional BF TEM images show numerous defects, twins and translation boundaries. However, careful electron microscopy diffraction observations have shown in various locations of the samples a few additional low-intensity spots, like the one located at 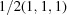, that are typical of a second unexpected phase. We identified this low-temperature phase as a monoclinic deformation of the orthorhombic phase appearing below 473 K. It results from a small shift of the atomic positions along the *z* direction for both Ni and Zr, and is analogous to the phase discovered several years ago by Bendersky *et al.* (1996 ▸) in the (Pd,Zr) system. It is discussed in more detail in Appendix *A* [App appa].

### 4.1. Quinary twins   

**Figure 7:**
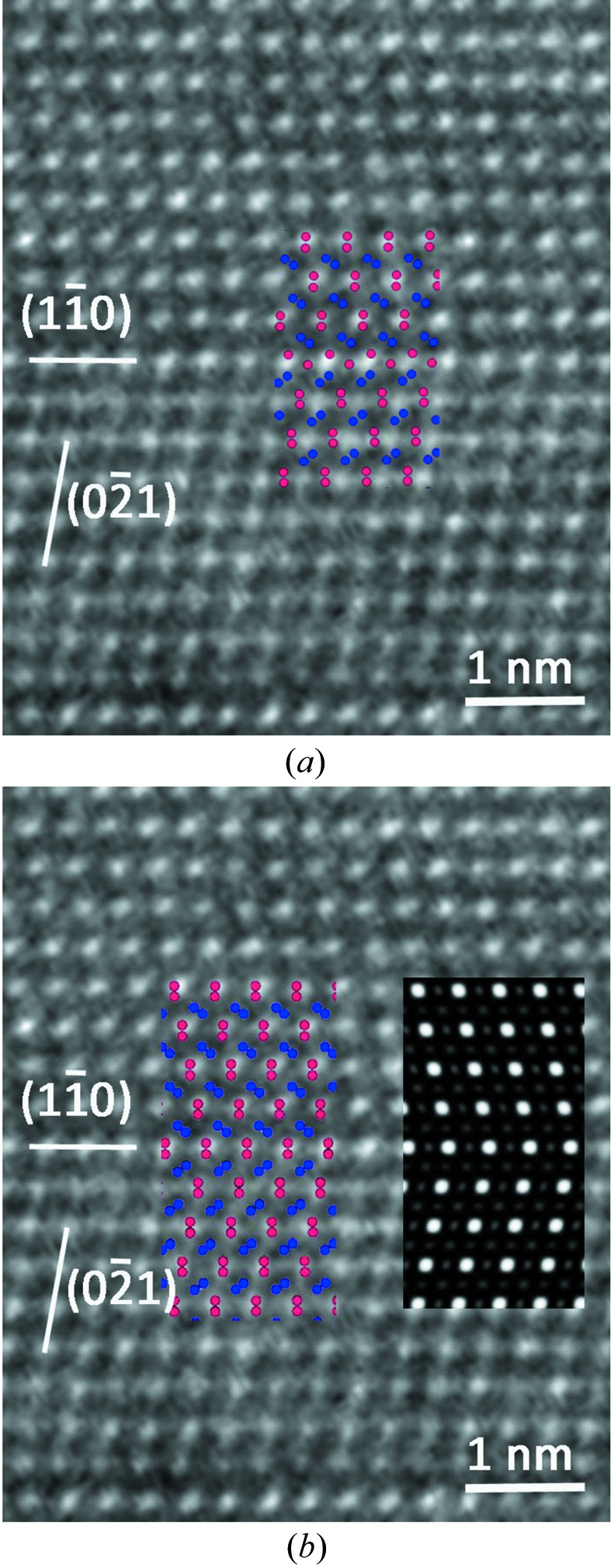
Quinary twin observed in HREM along the [112] direction with superimposition of the model 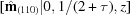 expressed in the *Cmcm* unit-cell coordinates shown in Fig. 4 ▸ with 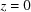 (*a*) and 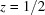 (*b*). The distribution of white dots in the micrograph at the level of the interface is clearly in favour of the 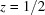 solution.

Quinary twins are very frequent in both samples (*a*) and (*b*) and quite easy to recognize. In order to make a full three-dimensional analysis of the associated translation, we made several HREM observations along the [112] direction to determine the translation part along the *z* direction. The result is seen in Fig. 7 ▸ together with the atomic simulations from our model with and without the 1/2 translation along *z*: it is clear that only the model with the *z* = 1/2 component corresponds to the experimental image.

**Figure 8:**
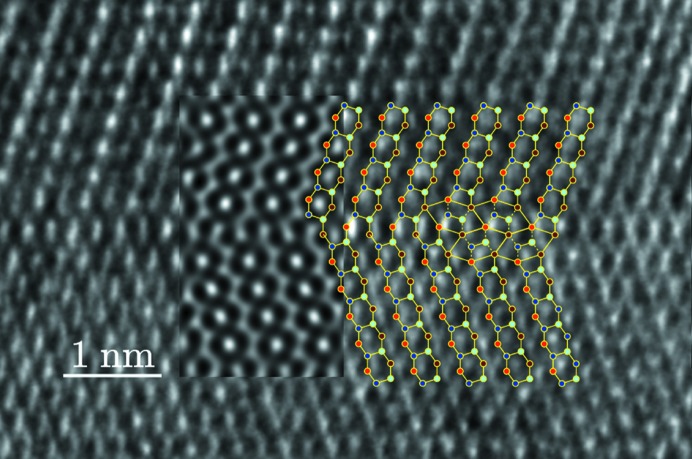
HREM image of a quinary twin observed along the [001] direction showing the translation associated with the mirror twin with the expected model and an insert of the image simulation made with the theoretical translation 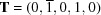 of our model.

In addition to these observations along the [112] direction, we performed HREM, STEM-BF and STEM-HAADF observations to determine the translation components in the [001] plane using samples (*b*). The HREM picture (CEMES) in Fig. 8 ▸ shows a clear translation associated with the mirror twin that fits quite well with the expected model of Fig. 4 ▸.

**Figure 9:**
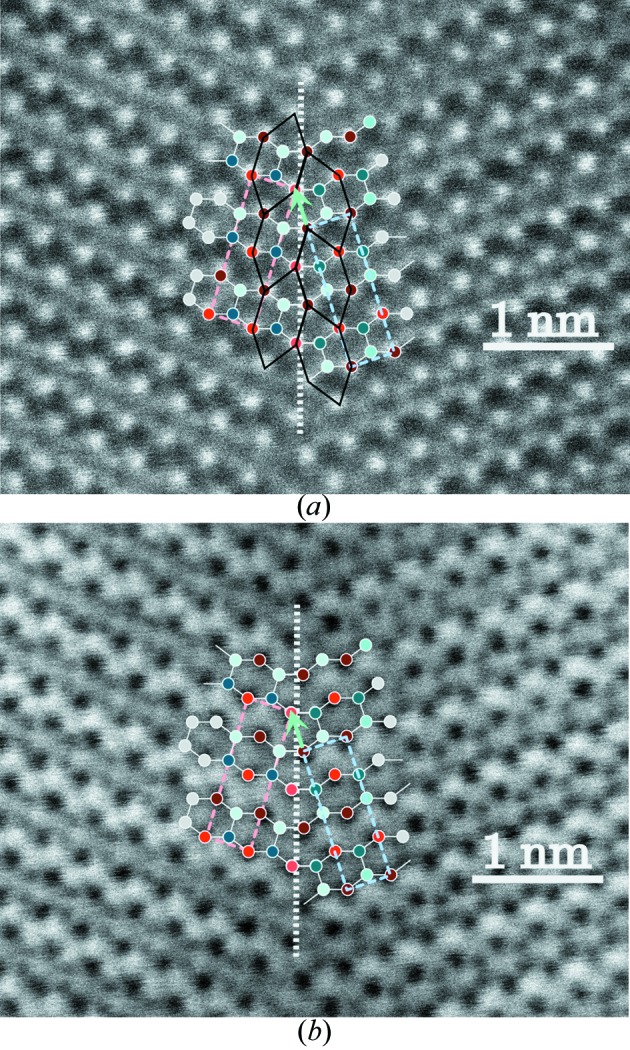
(*a*) STEM-HAADF atomic resolution of the quinary twin; (*b*) STEM-BF showing the remarkable invariance of the contrast on both sides of the boundary due to the very small local elastic field at the level of the boundary.

Observations at the ultimate resolution of atomic level in STEM-HAADF and STEM-BF modes performed on the same samples along the [001] direction fully confirm the model with a perfect agreement as shown in Fig. 9 ▸. The very comparable contrast variations at the level of the interface between the HAADF and BF micrographs prove that no significant displacements of the atomic positions occur at the boundary level owing to the remarkable crystalline coherency generated by the continuity of the tiling at the boundary crossing.

### 4.2. Translation defects and module dislocations   

**Figure 10:**
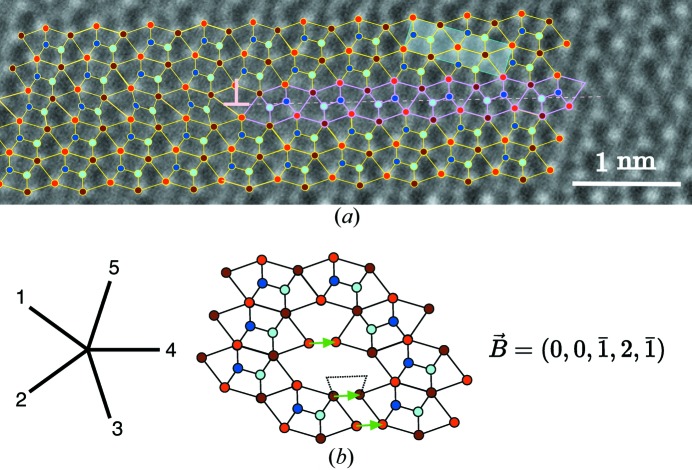
(*a*) HAADF observation of the elementary module dislocation defect in NiZr corresponding to the model of Fig. 6 ▸: the translation defect (in purple) with fault vector **T** = 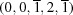 is bounded by the module dislocation with Burgers vector **B** = **T**. (*b*) Example of a circuit constructed on the 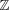-module determining the Burgers vector **B** = 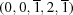 consistent with the translation vector **T** of the corresponding planar fault.

Here, too, the prediction of the model has been fully experimentally verified: the only translation boundaries we could observe were those generated by a single slab of microtwin embedded in the crystal corresponding to the models in Figs. 5 ▸ and 6 ▸. This is exemplified on the HAADF micrograph of Fig. 10 ▸(*a*) which shows the planar translation of the fault vector 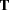 = 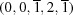 defect on the right, with the module dislocation of the Burgers vector 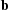 = 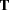 depicted in Fig. 10 ▸(*b*). The defect fits perfectly well with the simple theoretical model of Fig. 6 ▸.

The graphical way of determining the Burgers vector for an experimental picture is trivially achieved by reconstructing the very same sequence of undistorted hexagonal tiles as the one observed in the picture all around the defect and then measuring the closure default (in green in Fig. 10 ▸ *b*).

We have analysed many defects on the various samples and have always found that their configurations can be decomposed using our three basic defects: quinary twin, translation boundary and the corresponding elementary module dislocation.

**Figure 11:**
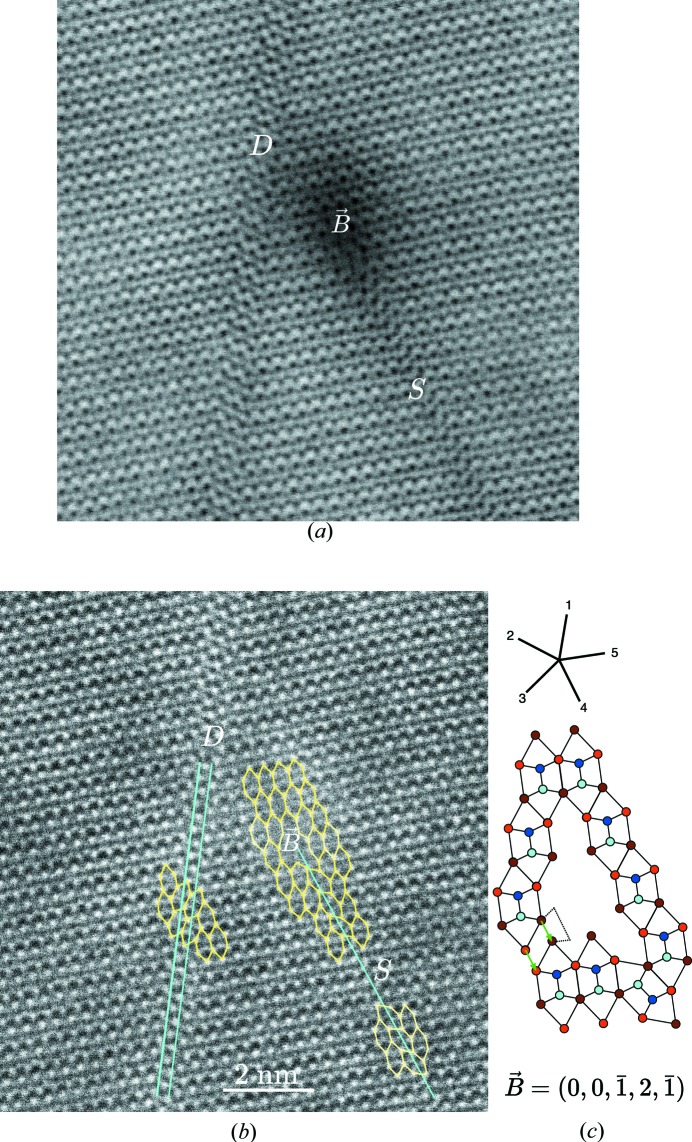
High-resolution STEM-BF (*a*) and STEM-HAADF (*b*) micrographs showing the two basic kinds of translation defects (*S* and *D*) and the elementary module dislocation *B*. The line noted *S* shows the simplest translation defects corresponding to the model in Fig. 5 ▸ made of one unique slab of hexagons with fault vector 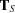 = 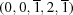. The double line *D* shows the translation defects generated with two slabs of hexagons 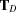 = 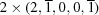. Finally, the dislocation *B* can be analysed using a simple generalization of Burgers circuit drawn in (*c*) leading, as expected, to a Burgers vector 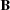 = 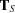 = 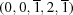. As shown in (*a*), the translation defects generate almost no local deformation in contrast to the dislocation *B* which is surrounded by an observable displacement field generating the dark shadow extending for a few atomic distances.

Fig. 11 ▸ shows at once three basic defects observed in both STEM-BF (*a*) and STEM-HAADF (*b*) modes. The translation boundary noted *S* is the exact experimental realization of the model of Fig. 5 ▸; the translation defect noted *D* is the two-slabs version of this defect. Finally, the localized defect noted *B* is the module dislocation that bounds the default *S*. A way of analysing this dislocation consists of drawing a close circuit of the basic hexagons around the defect (in yellow in Fig. 11 ▸ *b*) and reproducing the very same circuit using ideal undeformed hexagons (see Fig. 11 ▸ *c*): the closure defect is a direct measure of the Burgers vector. Here, we find 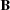 = 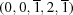 which is, as expected, the value of the translation 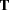 associated with *S*.

**Figure 12:**
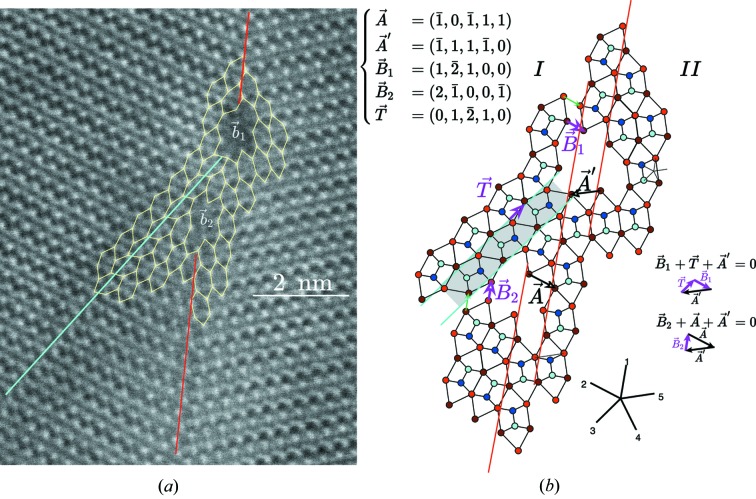
Two module dislocations, a translation boundary and a quinary twin. The translation boundary is along the line drawn in blue, whereas the quinary twin interface is along the red line(s). The twin boundary is displaced by one step **A** or equivalently **A**′ at the level of each module dislocation.

Many other more complex configurations have been observed. For example, Fig. 12 ▸ shows a HAADF picture with two module dislocations, one translation defect (in blue) and quinary twins (in red). We note by 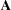 and 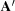 the unit vectors 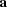 in, respectively, crystal I and II: 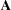 = 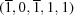 and 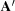 = 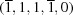. Having 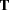 = 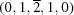 and using the Burgers circuit on the module drawn in Fig. 12 ▸(*b*) we obtain 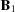 = 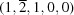 and 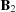 = 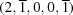. It is thus easily verified that 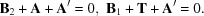
These relations are consistent with the fact that the twin boundary is displaced by one step along 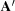 at the level of each module dislocation.

## 5. Discussion   

All ultra-high-resolution HAADF or BF images presented in this study are various realizations of a *single-module* crystal in the sense that the entire observation areas are described on a unique 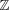-module that is invariant everywhere on the picture: vectors relating any two (equivalent) white dots on the micrographs are integer linear combinations of the five pentagon unit vectors with constant sum.

Some module dislocations are located inside the crystals (see Fig. 10 ▸), others are located at the interfaces between twinned crystals like those of Fig. 12 ▸. In both cases, these dislocations are the same type of module dislocations with the same Burgers vector of type 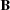 = 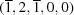 whatever their locations with respect to the other defects.

The use of 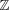-modules in crystallography goes beyond solely structural aspects. In fact, any geometrical description that deals with more than one crystal as in the case of the geometry of grain boundaries [see for instance, Pond (1989 ▸) and Hirth & Pond (1996 ▸)] is naturally adapted to the use of 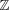-modules as a basic description tool. For example, let 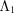 and 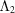 be the lattices of two adjacent crystals defined by the unit-cell vectors, respectively, 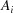 and 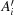, *i* running from 1 to 3. The natural module to consider is generated by the union 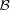 = 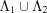, also called a bilattice, that is the set of the points ζ such that
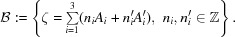
This module 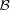 has a rank *N* between 6 and 3 according to the relative orientation of the lattices 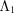 and 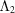. It is usually the optimized description of the bicrystal: an *N*-dimensional lattice representation that includes all possible kinds of defects that can be encountered in the study of the bicrystal.

However, in specific cases, this module might well not be the most appropriate to describe defects. Indeed, there are cases, as in NiZr, where the atomic structure itself is a decoration of a deeper hidden 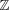-module generated by the *N* vectors 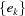. In that case, the unit cells 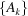 and 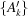 of the two lattices 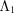 and 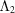 can be expressed as integer linear combination of the *N* vectors 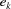: 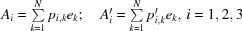
and thus 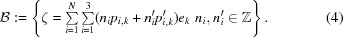
Thus, according to the values of the 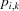 and 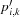, the set 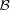 may define only a fraction of the 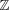-module.

**Figure 13:**
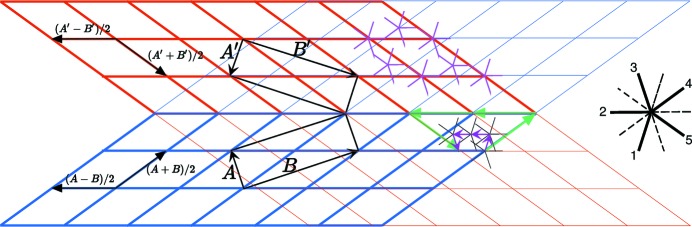
The bilattice 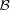, a module of rank 3 generated by the quinary twin in NiZr, is a subset of the pentagonal 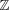-module. The basic module dislocation has Burgers vector 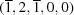 on the 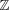-module (in purple) and 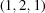 (in green) on 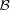. Here, the pentagonal module allows for a finer description of the possible interface dislocations than the standard three-dimensional module defining the bilattice.

For example, in the case of NiZr – and disregarding the direction *z* that is common to both twinned crystals – we have *A* = 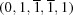, *B* = 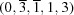 and 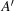 = 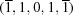, 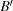 = 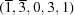 with a lattice of type *C*. The set 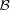 is thus defined by
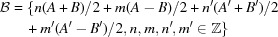
or using equation (4) explicitly 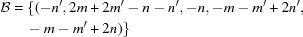
with 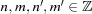. In the five-dimensional representation, this set of points is localized on a four-dimensional hyperplane perpendicular to the main five-dimensional diagonal 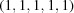 (the sum of the five components is zero whatever the values of 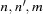 and 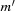). It has rank 3 because 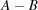 = 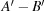, so that dislocations in that framework are characterized by three integer indices only. *A general module dislocation of the five-dimensional description might well not be in the set* 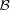. Such is the case for the hypothetical dislocation of Burgers vector 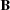 = 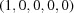 that does not belong to 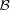. In contrast, the elementary dislocation of Burgers vector 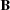 = 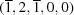 belongs to 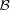 with *n* = 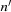 = 1, 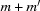 = 2, leading thus to 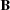 = (1, 2, 1) expressed in the three-dimensional module 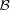. The two approaches are sketched in Fig. 13 ▸.

## 6. Conclusion   

This article is the experimental counterpart of a former one (Sirindil *et al.*, 2017 ▸) based on the idea of testing whether certain structures can be described in the context of 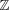-modules, *i.e.* in high-dimension spaces, rather than in the standard framework based on three-dimensional lattices. We have shown here that the orthorhombic phase NiZr can be faithfully described in a five-dimensional space with high internal symmetry generating possible defects at the symmetry breaking induced by the projection back in the three-dimensional space. The ultra-high-resolution electron microscopy pictures have shown perfect agreement between observed and predicted defects. This set of experiments supports the use of 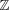-modules in crystallography; this is indeed an interesting and fruitful unifying concept, even in direct space, where it is both an elegant formulation and an efficient tool to predict new possible defects including interface dislocations in structures with hidden non-crystallographic symmetries, in a unique mathematical framework.

## Figures and Tables

**Figure 14:**
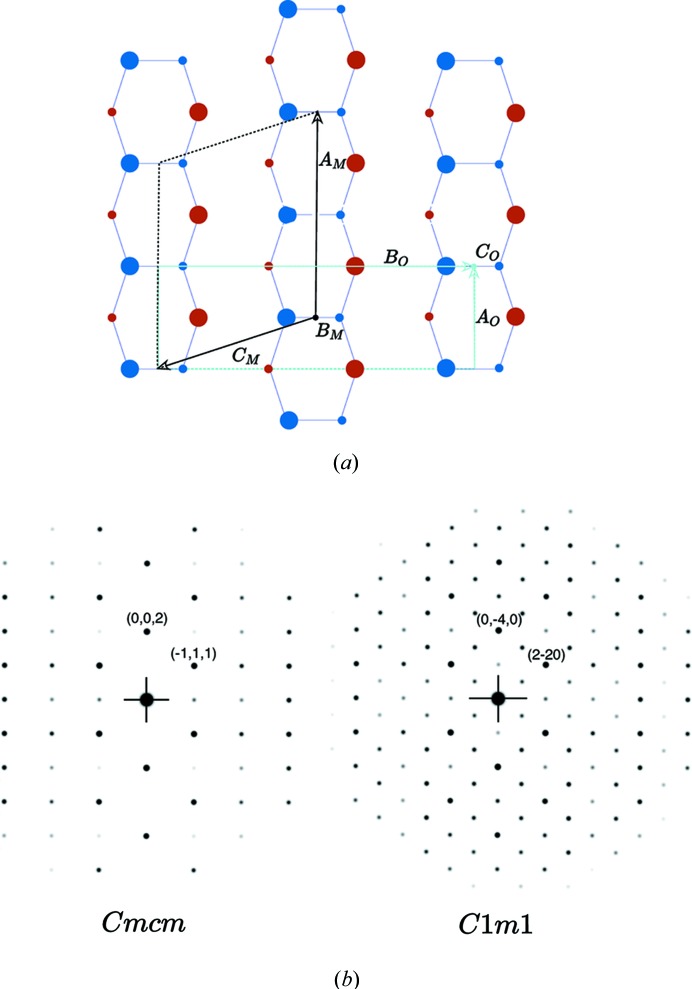
(*a*) The monoclinic *C*1*m*1 unit cell 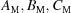, in black, and the orthorhombic *Cmcm* 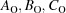, in light blue. (*b*) Corresponding diffraction patterns for both structures.

**Figure 15:**
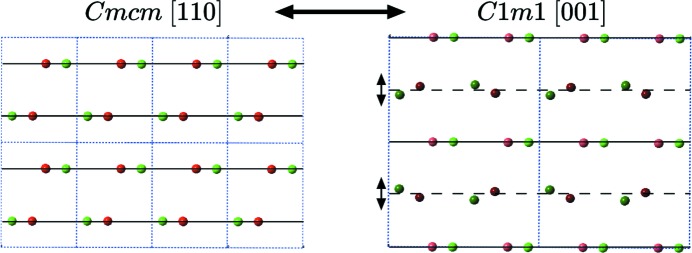
Ni are in red and Zr in green. In the monoclinic phase on the right, the atoms located on the glide mirror can move freely out of the mirror plane: the difference between the two structures consists of moving these atoms perpendicularly to the mirror.

**Figure 16:**
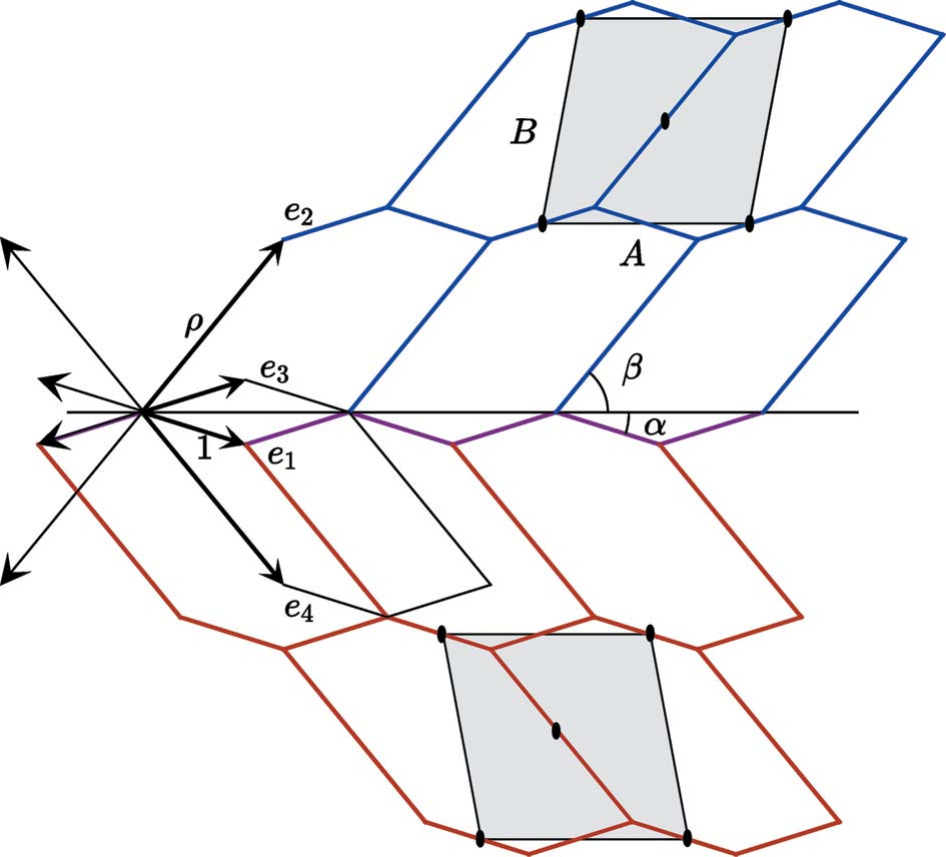
General coherent twin for structures defined by hexagons generated by 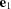, 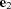 and 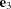 with 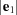 = 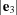 = 1 and 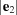 = 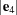 = ρ. The angles α and β are defined as 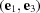 = 2α and 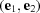 = 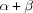. The structure has the space group *p*2 whereas the 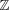-module is the projection of the four-dimensional lattice of space group *pmm*.

**Figure 17:**
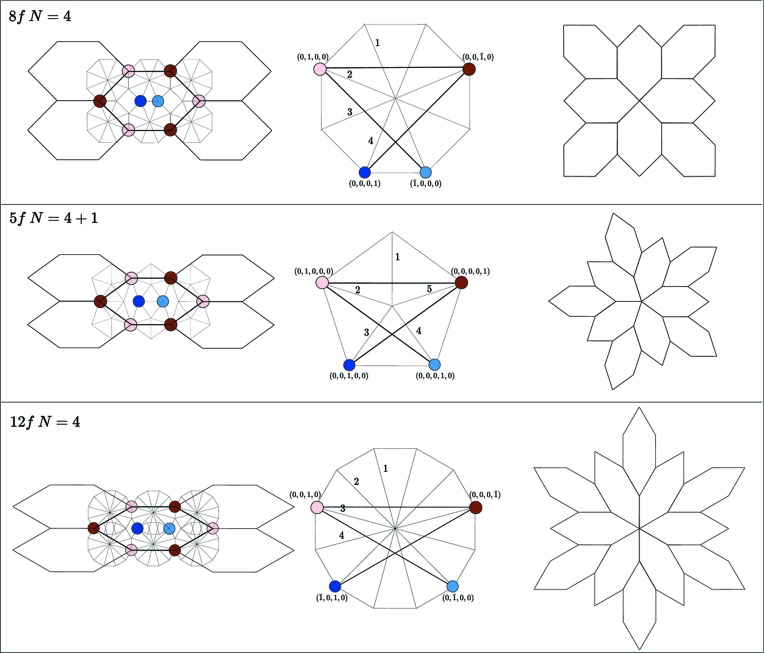
Interesting specific cases appear for the CrB-type structure where ρ = 1 if α = 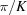, 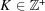. On top, the case *K* = 8 generates octagonal twins that are described using a 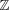-module of rank 4 defined by the four vectors 1, 2, 3 and 4 in the very same way as the pentagonal module (*K* = 10) of NiZr. The case of *K* = 12 leading to dodecagonal twins is also very simple to handle since the corresponding 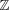-module is also of rank 4. Unit cells are: octagonal *A* = 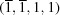, *B* = 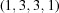; dodecagonal *A* = 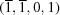, *B* = 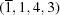 [see Hornfeck *et al.* (2014 ▸) Fig. 7 for an illustration of chiral twins of the CrB type with octagonal and dodecagonal symmetry].

## Footnotes

1 The accuracy of this ideal description is given by the ratio of the lattice parameters |*a*|/|*b*| = 0.3268/0.9973 = 0.3277 instead of the ideal value   0.3249 for perfect regular pentagons; this gives an accuracy with respect to the ideal model better than 1%.

2 In that regular pentagonal scheme, the ideal positions should be  =  =  = 0.08541 instead of 0.0817 and  =  =  = 0.3618 instead of 0.3609.

3 The translation orbit of a point  is the subset of its equivalent points that can be written as  where  is a translation of the lattice, irrespective of the point symmetry of the crystal.

4 Any translation  associated with a point operation  decomposes into a *reducible* part  that can reduce to zero by an *ad hoc* choice of origin, and an invariant *irreducible* part . This decomposition is particularly simple when  is of order 2; , since then  has eigenvalues . Thus,  is in the eigenspace associated with the eigenvalue +1 and  in the one associated with the eigenvalue −1: .

5 A typical ESL processing cycle with NiZr consists of the following steps: the levitating sample is heated with an infrared laser (*P* = 75 W, λ = 808 nm) in order to melt the sample; the temperature of the melt is further increased by about 50 K above  in order to evaporate possible contaminants. The heating laser is turned off, the melt cools and eventually undercools below  only through radiation of heat, with a mean cooling rate of about 50 K s^−1^; solidification sets in at Δ*T* = 300 K below . A NiZr sample, which solidifies at such undercoolings, exhibits two distinctive poles on its surface, similar to a globe. In the sample interior these poles are connected by the crystallographic direction [0,0,1] (see Hornfeck *et al.*, 2014 ▸). With these surface features one has the opportunity to orient the spherical sample and cut it perpendicular to [0,0,1], which was realized with a custom-built orientation tool.

## Appendix A The low-temperature monoclinic structure

We occasionally observed weak supplemental peaks at the positions 1/2(1, 1, 1) in the electron diffraction patterns with alternative strong and weak reflections typical of a slight deviation from the CrB-type ideal structure; this was demonstrated several years ago by Bendersky *et al.* (1996 ▸) in the case of the (Pd,Zr) system which is very similar to (Ni,Zr). A transformation appears below 473 K towards a monoclinic variant with space group *C*1*m*1 as shown in Fig. 14 ▸. The results of our own crystallographic analysis corroborate and complete the remarkable analysis of Bendersky *et al.*. The monoclinic phase is characterized by the unit-cell parameter ,  and  deduced from the orthorhombic parameters ,  and  according to

In reciprocal space, the relations are given by

leading to the following relationships between the indices:

The lattice indices are given by the absolute value of the determinant of the unit-cell relationships:

leading to the translation orbit decomposition (expressed on the *Cmcm* basis):

and the point group decomposition:

so that the complete basic coset decomposition for this transformation is written:

expressed on the *Cmcm* basis. This shows that, in the monoclinic phase, we should observe four types of orientational variants times two types of translational variants, a total of eight variants which all share the same reciprocal lattice for even *k*. This explains the complexity of planar defects observed by TEM compared with the relative simplicity of the diffraction patterns.

Initially described in *Cmcm* by the Wyckoff positions 4*c*:  and , for each Ni and Zr atomic species, the structure in *C*1*m*1 is described with the new Wyckoff positions

Explicitly for the present case , ,  so that we end up with the crystal description using the template^6^ for the two atomic species:

where we have translated the origin for *C*1*m*1 along the *b* axis by 1/8 to select it on the pure mirror perpendicular to the *b* axis (the *c* axis of the *Cmcm* structure). Here, β is equal to 1 in the orthorhombic structure and can take any value in the monoclinic one.

The diffraction amplitude  expressed in the reciprocal lattice of the monoclinic structure is easily calculated. Let us put :

or

with .

The first term vanishes for odd *k* and the second one vanishes for odd *k* and  (*i.e.* in the *Cmcm* structure). These extinctions are independent of the value of . This shows that the monoclinic structure is the derivation of the CrB-type structure where , *i.e.* where the atoms generated by  and  are (slightly) displaced along the *y* direction of the monoclinic phase, *i.e.* the *z* direction of the CrB-type structure.

In summary, the case  generates the diffraction pattern corresponding to the standard CrB *Cmcm* structure whereas  generates the diffraction corresponding to the monoclinic distorted structure with half of the atoms of Ni(Zr) (slightly) displaced along the *z* direction of the CrB structure as shown in Fig. 15 ▸.

The most important result with respect to the main purpose of the present article is twofold:

(i) Whatever structure, orthorhombic or monoclinic, is actually observed in the microscope, there are no differences in the HREM and HAADF images if observed along the [001] direction (orthorhombic indexing).

(ii) Whatever the actual value of the parameter β in the monoclinic phase, the five-dimensional scheme used here remains perfectly valid.

## Appendix B A generalization of coherent mirror twins

The case of the quinary twin generated by a  mirror can be generalized to many structures outside those of the CrB type. In fact, this happens each time the structure has the property of being possibly described as a two-dimensional tiling of identical hexagonal tiles where the hexagon has two adjacent sides of equal length as exemplified in Fig. 16 ▸ (Bouzy, private communication). There, the coherent twin is easily defined using the four-dimensional -module generated by the four vectors , ,  and , where  and  are mirrors of, respectively,  and . Choosing  as the length unit, we define ,  and . The two-dimensional unit cell is defined by  and  with space group *p*2. The projection matrix (not normalized) into the physical space is thus

The four-dimensional lattice generating the -module has space group  with *m* =  and *m*′ =  so that the coset decomposition of  onto *p*2 with the same lattice gives two variants, *i.e.* one twin characterized by the mirror  =  and its associated translation **t** decomposes into  =  =  and  = . The translation **t** can be expressed in the unit cell of the transformed variant as **t** = . Fig. 17 ▸ shows, beyond the pentagonal case, the two special high-symmetry cases of octagonal and dodecagonal systems, both modules of rank 4, where easy coherent twins are expected to occur.
